# Downregulation of LHCGR Attenuates COX-2 Expression and Induces Luteinized Unruptured Follicle Syndrome in Endometriosis

**DOI:** 10.3389/fendo.2022.853563

**Published:** 2022-05-04

**Authors:** Ting Geng, Yifan Sun, Lin Cheng, Yuming Cao, Ming Zhang, Zhidan Hong, Ling Ma, Yuanzhen Zhang

**Affiliations:** ^1^ Reproductive Medicine Center, Zhongnan Hospital of Wuhan University, Wuhan, China; ^2^ Hubei Clinical Research Center for Prenatal Diagnosis and Birth Health, Wuhan, China; ^3^ Department of Obstetrics and Gynecology, Zhongnan Hospital of Wuhan University, Wuhan, China

**Keywords:** endometriosis, luteinized unruptured follicle syndrome, LHCGR, ovulation, COX-2

## Abstract

An association between endometriosis and luteinized unruptured follicle syndrome (LUFs) has long been identified. Although inactivating mutation of luteinizing hormone/choriogonadotropin receptor (LHGCR) results in LUFs, whether LHCGR contributes to promoting LUFs in endometriosis remains elusive. To investigate the effect of LHCGR signaling in the development of endometriosis-associated LUFs and dissect the underlying mechanism *in vivo* mouse endometriosis model was established to measure the effect on ovarian folliculogenesis. *In vitro* cultures of primary human GCs collected from patients undergoing *in vitro* fertilization were performed and treated with human chorionic gonadotropin (hCG), dibutyryl cyclic-AMP (db-cAMP), LHCGR or CCAAT/enhancer binding protein-α (C/EBPα) small interfering RNA to identify the potential mechanisms. KGN cell line was used to investigate the mechanistic features of transcriptional regulation. Results showed an increased incidence of LUFs was observed in mice with endometriosis. The expression of LHCGR was decreased in the GCs of endometriosis mice. In *in vitro* cell models, LHCGR signaling increased the expression of C/EBPα and cyclooxygenase-2(COX-2), while inhibiting C/EBPα mitigated the induced COX-2 expression. Mechanically, C/EBPα bounded to the promoter region of COX-2 and increased the transcriptional activity under the stimulation of hCG or db-cAMP. Taken together, this study demonstrated that the LHCGR signaling was reduced in GCs of endometriosis and resulted in a decrease in gonadotropin-induced COX-2 expression. Our study might provide new insights into the dysfunction of GCs in endometriosis.

## Introduction

Endometriosis (EMs) is an estrogen-dependent chronic inflammatory condition that affects women in their reproductive period and causes infertility and pelvic pain. EMs has long been identified to have an association with luteinized unruptured follicle syndrome (LUFs), one of the ovulatory dysfunction subtypes, due to the intrafollicular endocrine milieu (1, 2). LUFs has been considered a subtle cause of endometriosis-associated infertility (3). The incidence of LUFs accessed by laparoscopic examination is 35% in endometriosis patients, while it is 11% in others (4). Increased incidence of LUFs is also observed in the animal model with endometriosis (5, 6). Although the mechanisms of ovulatory dynamics are similar to inflammatory responses (7), the precise underlying reasons for LUFs associated with endometriosis remain uncovered.

It has been observed that the dysregulation of follicle maturation and ovulation in endometriosis are tightly associated with endocrine and paracrine factors produced by granulosa cells (GCs) (8). The cyclooxygenase-2(COX-2)/prostaglandin E2 (PGE2), one of the major GCs derived factors, plays an essential role in the maintenance of normal oocyte maturation, follicle rupture and ovulation (9, 10). Moreover, COX-2 is aberrantly decreased in endometriosis, which may result in insufficient cumulus expansion and subsequently lead to impairment of the oocyte quality (11). However, the regulation mechanism of COX2 in endometriosis is largely unclear.

COX-2 is considered inducible by gonadotropin and participates in the regulation of reproduction, in addition, the luteinizing hormone (LH) surge regulates the expression of COX-2 and promotes biosynthesis of PGE2 within the ovulatory follicle (12). The biological activity of LH is mainly mediated by receptor-mediated signal transduction cascades and activated LH subsequently provokes the expression of numerous endocrine factors, either in ovarian granulosa or thecal cells. Therefore, changes in the LHCGR, which plays a vital role during ovarian development and corpus luteum function (13, 14) in women, might impact the correct course of these processes. Recent observations have demonstrated that abnormal LH signaling may be involved in the coexistence of anovulation and endometriosis (15). The clinical observation that patients with endometriosis have dysfunctional LHCGR expression (16), further suggesting a failure in the mechanism associated with LH action in the ovulation process. These observations led us to investigate whether LHCGR involved in COX-2 induced ovulation disorder.

Despite accumulating evidence exploring the mechanisms involving normal folliculogenesis and ovulation, the specific mechanism of LUF syndrome in endometriosis currently has not been elucidated. In this study, we found that LHCGR expression decreased in endometriosis granulosa cells. Functional studies in mice model and primary cultured granulosa cells revealed that attenuated LH signaling induced ovulatory disorder, mechanically, the inactivation of LHCGR induced decreased C/EBPα, which upregulated COX2 expression by binding to its promoter. Collectively, these results indicated that the decline of LHCGR may result in LUFs, and this may be associated with endometriosis-associated infertility.

## Materials and Methods

### Mice Model of Surgical-Induced Endometriosis

To improve our understanding of the pathophysiology underlying this enigmatic disease, animal models have been employed due to the ethical limitations of performing controlled studies of infertile women with endometriosis. The procedures on animals were carried out following institutional guidelines and the Institutional Animal Care and Use Committee of Wuhan University approved the experimental protocol (Approval No. WP2020-08005).

Five-week-old female C57BL/6 mice (Vital River Laboratory, China) were housed under well-controlled conditions (12 h light/12 h dark cycle maintained at a temperature of 22–25°C). After a week of acclimation, mice were injected with 17 β-estradiol (Sigma-Aldrich, E2758) (3 μg/mouse, s.c.) for 1 week, then the endometriosis model was conducted by autologous transplantation of uterine tissue (17). Briefly, after euthanized, the left uterine horns were isolated. Obtained uterine tissue was cut into three equal-sized parts as implants, auto-transplanted was performed around three arteries of the intestinal mesentery. Sham-operated control mice (sham) were subjected to the same steps, but no implant was sutured to the intestinal mesentery. To allow the recuperation and development of endometriotic implants, the subsequent experiment began after 3 weeks.

### Superovulation, Oocyte Collection and Sample Harvest

After 3 weeks, mice were superovulated with 5 IU pregnant mare serum gonadotropin (PMSG) (Solarbio, P9970) followed 48 h later by 5 IU human chorionic gonadotropin (hCG) (LIVZON, China) to induce follicle development and ovulation. When mimicking the poor response to LH surge of ovary *in vivo*, the mice were treated with full-dose PMSG (5IU) followed by half-dose hCG (2.5 IU) to trigger ovulation. Ovarian tissues for follicular morphology were collected before superovulation or 48 h after PMSG administration. Granulosa cells for gene expression analysis were isolated at different times after hCG administration (0, 2, 4, and 8 h). To exam the number of ovulated oocytes, ampullae were collected at 14–16 h after hCG injection and then secured to release the clutch of cumulus–oocyte complexes (COCs). For morphology analysis of the post-ovulatory ovary, the samples were collected at 24 h after hCG administration.

### H&E Staining and Immunohistochemistry

For follicle counting, ovaries from each group were collected at 14–16 h after hCG injection and hematoxylin and eosin (H&E) staining was performed as described previously (18). Briefly, the right ovaries were fixed in 4% paraformaldehyde, routinely paraffin-embedded, then cut thoroughly into sections of 5-μm thickness followed by staining. In every fifth section, follicles containing oocytes with a visible nucleus were counted and properly classified into different follicle stages (19). The number of luteinized unruptured follicle and corpus luteum (CL) were also recorded.

Mice ovaries were collected as mentioned above and Immunohistochemistry was performed on formalin-fixed paraffin-embedded sections using immunoperoxidase staining kit (PV-9001, ZSGB-BIO, Beijing, China) according to the procedure of the manufacturer. After deparaffinized and rehydrated, antigen retrieval was carried out with sodium citrate. Approximately 3% hydrogen peroxide was used to eliminate the activity of endogenous peroxidase. The sections were then treated with bovine serum albumin (BSA) blocking, followed by incubation with primary antibody for LHCGR (19968-1-AP; 1:200 dilution; Proteintech), C/EBPα (18311-1-AP; 1:200 dilution; Proteintech), COX-2 (ab15191; 1:200 dilution; Abcam) and corresponding secondary antibody. The omission of the primary antibody served as a negative control. The H-score was processed and calculated as described previously (20). We used the following equation: H-score = ∑ Pi (i), where i was the intensity of staining with a value of 1, 2, or 3 (weak, moderate, or strong, respectively) and Pi was the percentage of stained cells for each intensity, varying from 0 to 100%.

### Human Granulosa Cell Collection and KGN Cell Line Culture

The study protocol was approved by the Institutional Review Board (No. 2018047). Human GCs were obtained from patients undergoing *in vitro* fertilization (IVF)/intracytoplasmic sperm injection (ICSI) treatment due to tubal factor or male subfertility at the Reproductive Medical Center of Zhongnan Hospital. After controlled ovarian hyperstimulation, 10,000 IU hCG was administered to trigger ovulation. The follicular fluid was immediately collected and centrifuged for 10 min at 2,000 rpm after oocyte pick-up. Then the pellet was resuspended in an enzymatic solution to digest clusters of cells. GCs were highly isolated through Percoll density gradient and red blood cells were removed using lysis buffer. The cell pellet was resuspended in DMEM/F12 medium (Gibco) supplemented with100 U/ml penicillin, 100 µg/ml streptomycin, and 10% (v/v) fetal bovine. The cells were then seeded at a density of 2 × 10^5^ cells/ml in a 6-well plate and incubated for 3 days at 37°C in humidified atmosphere with 5% CO_2_. The media was replenished every 24 h. To mimic the effect of LH and cAMP *in vivo*, we used hCG (10 IU/ml; LIVZON, China) and dibutyryl-cAMP (db-cAMP, 1 mM; HY-B0764A, MedChemExpress, USA) respectively to stimulate cells and further cultured for stated hours *in vitro* according to previous studies (21). KGN cell line was cultured in DMEM/F12 medium as mentioned above.

### Small Interfering RNA (siRNA) and Transfection

For gene silencing experiments, human GCs were transfected with 50 uM small interfering (siRNA) oligonucleotides against LGCGR, C/EBPα or negative control (NC) siRNA (Huzhou Hippo Biotechnology Co., Ltd.) using lipofectamine 3000 transfection reagent (Invitrogen, USA) according to the instructions provided by the manufacturer. The specific sequences of target genes were as follows: si-LHCGR, 5′-UGC CUU CAA AGU ACC UCU UAU TT-3′ (sense) and 5′-AUA AGA GGU ACU UUG AAG GCA TT-3′ (antisense); si-C/EBPα, 5′-GGA GCU GAC CAG UGA CAA UTT-3′ (sense) and 5′-AUU GUC ACU GGU CAG CUC CAG-3′ (antisense); si-LHCGR scrambled NC, 5′-GUC AUU AUC CUU UCG CAC UAA dTdT-3′(sense) and 5′-UUA GUG CGA AAG GAU AAU GAC dTdT-3′ (antisense); si-C/EBPα scrambled NC, 5′-GGU AAC GGG ACC GAC UUA AdTdT-3′ (sense) and 5′-UUA AGU CGG UCC CGU UAC CdTdT-3′ (antisense). For further experiments which were focused on the mechanisms of signal pathways, the cells were incubated with or without db-cAMP or hCG for further 24 h after 24 h of transfection (22).

### RNA Isolation and Quantitative Real-Time PCR (qRT-PCR)

Total RNA was extracted with an RNA extraction kit (RN0302, Aidlab, China). RNA (1 ug) was reverse transcribed with a cDNA Synthesis Kit (R212-01, Vazyme, China). Quantitative real-time PCR (qRT-PCR) was performed using ChamQ SYBR qPCR Master Mix (Q311-02, Vazyme, China) and a CFX96 PCR system machine (Bio-Rad Laboratories, USA). Each reaction was performed with a total volume of 20 μl, consisting of 2× ChamQ SYBR qPCR Master Mix (10 µl), 5’- and 3’-primer (0.4 µl, respectively), cDNA (1 µl), and ddH2O (8.2 µl). With the following primers: LHCGR: 5′-TCC TTT CCA GGG AAT CAA TC-3′ (sense) and 5′-GGC CGG TCT CAC TCG AC-3′ (antisense); C/EBPα: 5′-CAC GAA GCA CGA TCA GTC CAT-3′ (sense) and 5′-CGG AGA GTC TCA TTT TGG CAA G-3′ (antisense); COX-2: 5′-TAA GTG CGA TTG TAC CCG GAC-3′ (sense) and 5′-TTT GTA GCC ATA GTC AGC ATT GT-3′ (antisense); GAPDH: 5′-CTG TTC GAC AGT CAG CCG CATC-3′ (sense) and 5′-GCG CCC AAT ACG ACC AAA TCC G-3′ (antisense). Data analysis was performed using Bio-Rad CFX manager system, using GAPDH as a reference transcript.

### Western Blot Analysis

Whole-cell protein extract was lysed and isolated from cultured cells or mouse ovaries. After measuring protein concentrations using a BCA Protein Assay Kit (P0010, Beyotime, China), equal amounts of denatured protein were separated by electrophoresis in 10% SDS polyacrylamide gels and transferred to polyvinyl difluoride membranes (Millipore, Billerica, USA), which were then saturated with blocking buffer for 1 h. After that, the membranes were incubated with rabbit polyclonal anti-LHCGR (19968-1-AP; 1:1,000 dilution; Proteintech), rabbit polyclonal anti-CEBPα (8178; 1:1,000 dilution; Cell Signaling), or rabbit polyclonal anti-COX2 antibodies (ab 15191; 1:1,000 dilution; Abcam) O/N at 4°C. The blots were incubated with HRP–conjugated anti-rabbit IgG for 1 h. Peroxidase activity was detected using the ECL system (Touch Imager, e-Blot, China).

### Immunofluorescence

After treatment for stated hours *in vitro*, cells were fixed with paraformaldehyde. Then, the cells were treated with 0.5% Triton solution to encourage intracellular labeling. Cells were next blocked with 5% BSA for 1–2 h at RT and incubated with primary antibody against CEBPα (8178; 1:200 dilution; Cell Signaling) O/N at 4°C. Secondary antibody incubation was performed at RT for 60 min. Cells were washed and treated with antifade mounting medium, which contains DAPI (P0131, Beyotime, China).

### Luciferase Reporter Assays

The effect of C/EBPα on the transcriptional activity of COX-2 promoter was determined by analyzing the dual-luciferase activities using a commercial assay kit (E2920, Promega, USA). The expression construct used for luciferase-based assays was pcDNA3.1 C/EBPα (NM_004364) while reporter construct used was pGL3-PTGS2 promoter (−1.2 kb/+137). KGN cells were co-transfected with the indicated plasmids with the help of lipofectamine 3000 transfection reagent (Invitrogen, USA) as previously described (23). The samples were lysed at 48 h after transfection, detection of luciferase activity was conducted. Firefly luciferase measurements were normalized to Renilla luciferase.

### Chromatin Immunoprecipitation Assay

After treatment with hCG or db-cAMP, KGN cells (2 × 10^7^) were collected and processed as described previously (24). Briefly, cells were washed and fixed in 1% formaldehyde for 15 min then cross-linking was terminated using 0.125 M glycine. Then the chromatin immunoprecipitation (ChIP) assay was performed to determine whether CEBPα interacts with the putative binding site in COX-2 promoter using a Simple ChIP Kit (56383, Cell Signaling) according to the protocols of the manufacturer. Approximately 1% of the chromatin fragments were stored at −20°C to be used later for input for normalization. For each immunoprecipitation (IP) reaction, every 5 ug chromatin sample was incubated with 4 ug CEBPα antibody (18311-1-AP, Proteintech) O/N at 4°C or with 1 ul IgG antibody (2729, CST) as a negative control for nonspecific IP. The primers for the COX2 promoter used in ChIP-PCR analyses were as follows: 5′-TCTAGGAAGCCTTTCTCCTCCT-3′ (sense) and 5′-TGATCCACGCTCTTAGTTGAAAT-3′ (antisense). The resulting signals were normalized to input values, with the IgG-negative control values subtracted as background.

### Statistical Analysis

Data were calculated as percentages or ratios relative to the corresponding negative controls, presented as means ± SEM, and were appropriately analyzed by ANOVA, or unpaired *t-*test with GraphPad Prism (Version 8.1.1, California). Values of *P <*0.05 were considered statistically significant.

## Results

### The Presence of LUFs in Mice With Surgical-Induced Endometriosis

**Figure 1:**
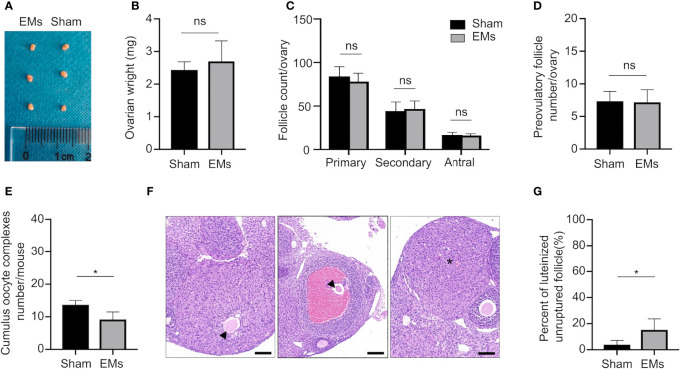
EMs mice with ovulatory dysfunction and LUFs. **(A, B)** Ovarian morphology and weights of EMs and sham mice. ns, no significance. **(C)** Average number of each follicle classes per ovary (every fifth section of serially sectioned ovaries was counted; n = 6, each group). ns, no significance. **(D)** Number of preovulatory follicles per ovary (n = 6, each group). **(E)** Number of ovulated oocytes per mouse (n = 6, each group). *P < 0.05 (Student’s *t*-test). ns, no significance. **(F)** Representative H&E-stained ovarian tissue sections depicting unruptured follicle and normal CL. Asterisk denoted normal CL after ovulation. Arrows pointed to the trapped oocytes within CLs. Scale bars, 100 μm. **(G)** The percentage of luteinized unruptured follicle for all CLs. The values were the mean ± SEM. *P < 0.05 (Student’s *t*-test).

After confirming the induction of surgical-induced endometriosis three weeks after the operation, we initially collected the ovaries and evaluated the impact of endometriosis on the general morphology and ovarian reserve (**Figures 1A, B**). The anatomical observation indicated both morphology and ovarian weight were similar between EMs and sham mice. Counts of primary, secondary and antral follicles in the endometriosis model (78.00 ± 9.72, 46.67 ± 9.14, and 16.00 ± 2.00, respectively) were comparable to those of sham-operated mice (84.00 ± 11.24, 44.33 ± 10.33, and 16.67 ± 2.94, respectively) (**Figure 1C**). These results suggested that endometriosis mice presented similar healthy follicles to sham mice.

Ovarian responsiveness to gonadotropins was further evaluated to examine if folliculogenesis and ovulation are affected by endometriosis. Firstly, ovaries were collected 48 h after PMSG treatment when follicles developed to preovulatory stage (**Figure 1D**). Quantification of ovarian follicles indicated that the number of preovulatory follicles showed no significant differences in animals of both groups (7.33 ± 1.50 in sham vs. 7.17 ± 1.94 in EMs). Subsequently, the number of ovulated oocytes was assessed after a superovulation protocol to further investigate whether ovulation was affected by endometriosis (**Figure 1E**). After 16 h of hCG administration, fewer cumulus–oocyte complexes (COCs) were released in EMs mice compared to that in sham-operated mice (9.12 ± 2.31 in EMs vs. 13.67 ± 1.37 in sham), indicating the ovulatory capacity was compromised in endometriosis mice.

During ovulation, the follicle ruptures and oocyte is released, the remaining GCs and theca cells under the influence of LH are luteinized to form a corpus luteum (CL). EMs ovaries showed increased luteinized unruptured follicles (**Figures 1F, G**), characterized by the oocytes destined for ovulation becoming entrapped in preovulatory follicles or corpora lutea within a full investment of luteinized granulosa cells. These observations established that, in endometriosis, LUFs leads to reduced ovulation and abnormal CL formation, and it may be a cause of endometriosis-associated infertility.

### Attenuated Responsiveness of LHCGR to its Ligand Leads to LUFs in Endometriosis

**Figure 2:**
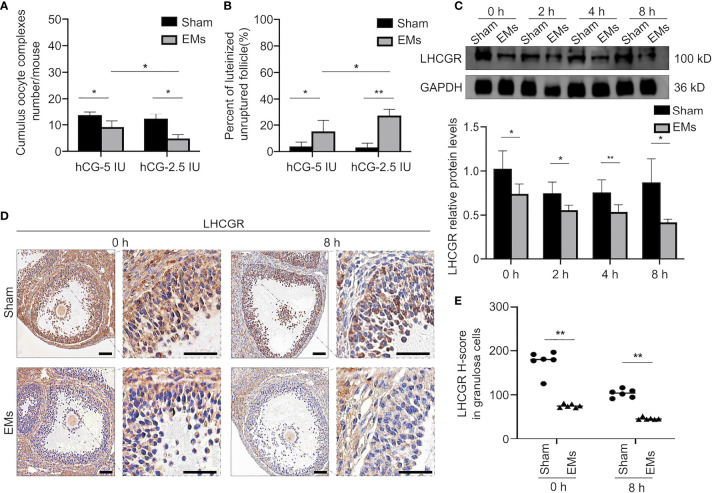
Decreased responsiveness of LHCGR to its ligand in endometriosis. **(A)** Average number of ovulated oocytes following superovulation protocols with a different dose of hCG (n = 6, each group). *P < 0.05 (two-way ANOVA). **(B)** The percentage of luteinized unruptured follicle for all CLs tracked per ovary. *P < 0.05, **P < 0.001 (two-way ANOVA). **(C)** Western blotting of LHCGR protein expression in GCs of EMs and sham mice at different time points after hCG administration (0, 2, 4, and 8 h). GAPDH was used as a sample loading control. * *P* < 0.05, **P < 0.001 (Student’s *t*-test). **(D, E)** Immunohistochemical H-score and representative images of immunohistochemical staining for LHCGR in the GCs from EMs and sham mice after PMSG-priming (48 h). Scale bar, 100 μm. **P < 0.001 (Student’s *t*-test).

To uncover the mechanism responsible for LUFs, we mimicked the poor response to LH surge of ovary *in vivo* by treating the mice with full-dose PMSG (5I U) followed by half-dose hCG (2.5 IU) to trigger ovulation. Although similar trends had been observed in full-dose hCG mice occurred in sham mice, the EMs mice displayed a significant reduction of ovulatory oocytes and more frequent incidence of LUFs after administration of half dose of hCG *in vivo* (**Figures 2A, B**). Moreover, the expression pattern of LHCGR protein in the GCs collected at different time points after hCG administration was determined by Western blotting (**Figure 2C**). The results showed that LHCGR protein levels were lower in the GCs of EMs mice in the early ovulatory phase compared with controls, the reduced trends even lasted to late ovulatory phases. IHC staining confirmed that the intensity of LHCGR staining appeared weaker and sporadic in GCs of hCG-primed (0 and 8 h) EM mice than that in sham mice (**Figures 2D, E**). These results suggested that decreased LHCGR induces a poor response to LH surge and therefore contribute to the pathogenesis of LUFs. More importantly, the dysfunction presents before endogenous LH surge.

### LHCGR Modulates COX-2 Expression in Human Granulosa Cells

**Figure 3:**
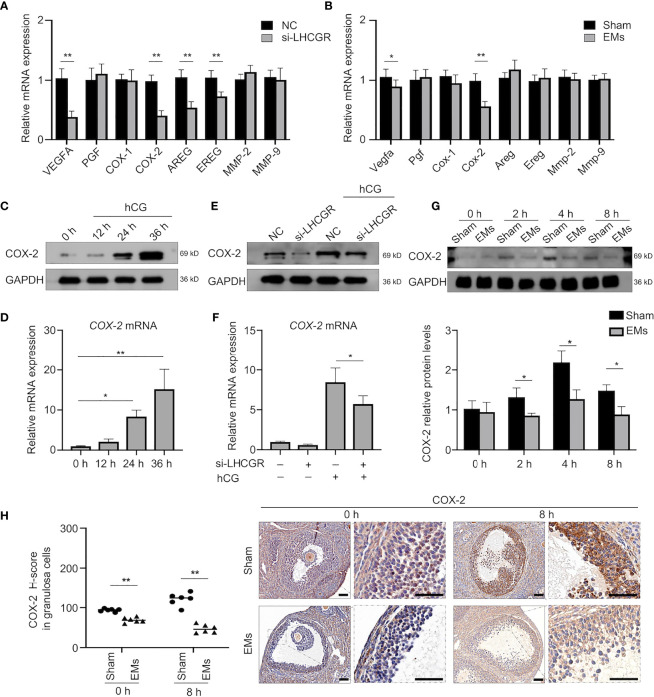
COX-2 involved in the hCG-induced ovulation is downregulated in EMs granulosa cells. **(A)** qRT-qPCR analysis of expression of ovulation-related genes VEGFA, PGF, COX-1, COX-2, AREG, EREG, MMP-2, and MMP-9 in human GCs treated with nontargeting negative control siRNA (NC) or LHCGR siRNA.**P < 0.001 (Student’s *t* -test). **(B)** qRT-PCR analysis of ovulation-related gene expression in GCs of EMs and sham mice after PMSG-priming (48 h). *P < 0.05, **P < 0.001 (Student’s *t* -test). **(C)** Human GCs were treated with10 IU/ml hCG for 0, 12, 24, and 36 h, the protein levels of COX-2 were examined by Western blot. **(D)** The mRNA levels of COX-2 in hCG-treated human GCs at different time points were analyzed by qRT-PCR. *P < 0.05, **P < 0.001 (ANOVA). **(E, F)** Human GCs were transfected with 50 nM siRNA against LHCGR for 24 h and then treated with 10 IU/ml hCG for another 24 h. The mRNA and protein levels of COX-2 were analyzed. *P < 0.05 (ANOVA). **(G)** Western blotting of COX-2 during ovulation in GCs from EMs and sham mice.*P < 0.05 (Student’s *t* -test). **(H)** Immunohistochemical H-score and representative images of immunohistochemical staining for COX-2 in the GCs from EMs and sham mice after PMSG-priming (48 h). Scale bar, 100 μm. **P < 0.001 (Student’s *t* -test).

LH signaling primes many key ovulatory genes in granulosa cells. To further look into the mechanism responsible for LUFs, we first investigate whether LHCGR is involved in the expression of ovulation-related genes (25–27). The qRT-PCR analysis indeed showed that the expression levels of known genes, such as VEGFA, COX-2, AREG, and EREG were significantly diminished in the human GCs of LHCGR knockdown than those of the negative control (NC) (**Figure 3A**). We further analyzed the gene expression between EMs and sham mice. Both VEGFA and COX-2 were significantly decreased in the GCs of EMs mice (**Figure 3B**). Since abnormal COX-2 function is also associated with ovulation failure (28), these observations led us to investigate the correlation between LH signaling and COX-2.

To determine the effect of LH signaling on COX-2, human GCs were stimulated with 10 IU/ml hCG to mimic the *in vivo* induction. The expression pattern of COX-2 in the human GCs collected at different time points after hCG administration was determined (**Figures 3C, D**). As expected, hCG treatment induced the expression of COX-2 in levels of mRNA and proteins at 24 h, and the levels remained appreciable even at 36 h after hCG treatment (**Figures 3C, D**). To reveal the functional role of LHCGR involved in the expression of COX-2 during the periovulatory period, RNA interference (RNAi) approach was employed to knock down LHCGR transcripts in the presence or absence of hCG. The knockdown of LHCGR *per se*, rather than negative control, recapitulated the hCG induced COX-2 upregulation (**Figures 3E, F**).These results revealed that LHCGR is involved in the hCG-induced upregulation of COX-2 expression in human GCs.

We further assess the expression of COX-2 during ovulation, granulosa cells were collected at different time points after hCG (0, 2, 4, and 8 h) treatment for analysis (**Figure 3G**). COX-2 protein of the granulosa cells from EMs mice were significantly decreased compared to sham mice. IHC staining for COX-2 showed that COX-2 was mainly localized to granulosa and theca cells of dominant follicles during both early and late ovulatory phases, and abundant COX-2 was found at 8 h after hCG priming, whereas little staining was observed in EMs mice (**Figure 3H**). Therefore, we conjecture that the endometriosis-related abnormal actions of LHCGR modulate downregulation of COX-2 in GCs, then results in reduced ovulation with impaired follicle rupture.

### LHCGR Regulates COX-2 Expression Through C/EBPα Protein

**Figure 4:**
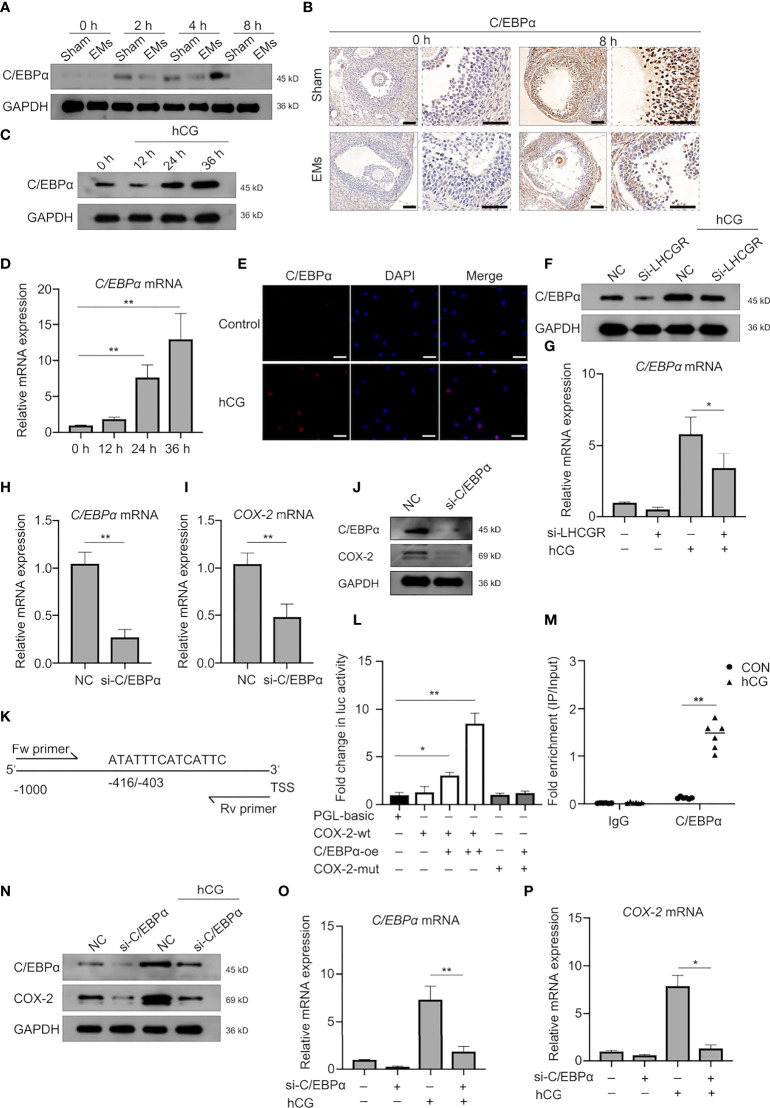
C/EBPα is necessary for hCG-induced COX-2 expression in granulosa cells. **(A)** Western blotting of C/EBPα during ovulation in GCs from EMs and sham mice. **(B)** Representative images of immunohistochemical staining for C/EBPα in the GCs from EMs and sham mice after PMSG-priming (48 h). Scale bar, 100 μm. **(C, D)** The protein and mRNA levels of C/EBPα in hCG-treated human GCs at different time points were analyzed by Western blot and qRT-PCR, respectively. **P < 0.001 (ANOVA). **(E)** hCG-treated (24 h) human GCs were analyzed by immunofluorescence to identify the subcellular localization and protein expression levels of C/EBPα (red). Nuclei were stained with DAPI (blue). Magnification: ×100. Scale bar, 50 μm. **(F, G)** Human GCs were transfected with negative control siRNA or LHCGR siRNA and then treated with hCG. The mRNA and protein levels of C/EBPα were analyzed. NC, negative control. *P < 0.05 (ANOVA). **(H–J)** Human GCs were transfected with negative control siRNA or C/EBPα siRNA. The expression of indicated genes and protein was analyzed by qRT-PCR and Western blot. **P < 0.001 (Student’s *t* -test). **(K)** Predicted C/EBPα-binding site in the promoter region of human *COX-2* . TSS, transcriptional start site; Fw primer, forward primer; Rev primer, reverse primer. **(L)** KGN cells were cotransfected with C/EBPα-overexpressing plasmid vectors, and luciferase reporter constructs harboring the COX-2 promoters, along with a Renilla luciferase construct for internal control. Firefly luciferase (Luc) activity was normalized to Renilla activity. Data are shown as mean ± SEM and expressed as fold increase in firefly luciferase activity compared with empty vector (PGL-basic). *P < 0.05, **P < 0.001 (Student’s t-test). **(M)** KGN cells were left untreated or stimulated with hCG for 24 h. ChIP assays were performed using anti-C/EBPα antibody or isotype control antibody (IgG). qRT-PCR was used to determine C/EBPα occupancy at the potential biding site under the conditions tested. **P < 0.001 (Student’s t-test). **(N–P)** Human GCs were transfected with negative control siRNA or C/EBPα siRNA and then treated with hCG. The expression of indicated genes and protein was analyzed by qRT-PCR and Western blot. **P < 0.001 (ANOVA).

We further sought to identify underlying mechanisms of the LHCGR-induced COX2 upregulation. There is evidence indicating the expression and functional activation of C/EBP family members is essential for events associated with reproduction (29, 30). We reasoned that C/EBPα may participate in the LHCGR-induced COX2 expression. Accordingly, Western blot was conducted to investigate the expression pattern of C/EBPα in granulosa cells collected at different time points after hCG administration and the results showed that C/EBPα was diminished in EMs mice (**Figure 4A**). IHC staining showed that, after PMSG administration (48 h), follicles were either mature or in the process of ovulation, C/EBPα was expressed in the granulosa and theca cells of superovulated mouse ovaries. After treatment with hCG, the protein level was significantly increased. Interestingly, C/EBPα staining in the EMs mice appeared to be attenuated in the granulosa cells (**Figure 4B**).

The results demonstrated that hCG escalated the expression of C/EBPα in granulosa cells after 24 h in both mRNA and protein levels (**Figures 4C, D**). We also confirmed that hCG significantly induced C/EBPα expression in the nuclei of human GCs (**Figure 4E**).

Consistently, knockdown of LHCGR affected the basal levels of C/EBPα expression; it also further significantly diminished the hCG-induced C/EBPα expression (**Figures 4F, G**). These results provided evidence that C/EBPα may involve in regulation by LHCGR signaling in human GCs. To further substantiate our observation, siRNA-mediated down-regulation of endogenous C/EBPα was employed, and we found that the expression of COX-2 was down-regulated after knockdown endogenous C/EBPα in GCs (**Figures 4H–J**).

To interrogate the bona fide regulation of C/EBPα on COX-2, dual-luciferase reporter assay using KGN cells was conducted and the results showed that C/EBPα was sufficient to operate as a transactivator of COX-2 transcription since the luciferase activity of cells transfected with COX-2 wild-type reporter plasmid (COX-2-wt) was strongly improved after co-transfected with C/EBPα-overexpression (C/EBPα-oe) plasmid in a dose-dependent manner (**Figure 4L**). These results indicated that C/EBPα activated the transcription of COX-2. To address the potential binding region of C/EBPα in the COX-2 promoter region, a bioinformatics analysis was conducted by JASPAR database (http://jaspar.genereg.net/), a putative C/EBPα-binding site located at position −416/−403 of the COX-2 promoter was identified (**Figure 4K**). The results of dual-luciferase reporter assay verified that the luciferase activity in cells co-transfected with COX-2 mutant reporter plasmid (COX-2-mut) and C/EBPα-oe plasmid was not altered (**Figure 4L**). Furthermore, ChIP-PCR assays were conducted to validate the molecular interaction between C/EBPα and the identified biding site in COX-2 promoter in KGN cell line collected 24 h after treatment with hCG (**Figure 4M**). The result unveiled that the immunoprecipitation of the C/EBPα antibody-enriched DNA fragments containing identified binding sites, demonstrating CEBPα was strongly bound to the promoter region upstream from the transcriptional start site of COX-2 gene.

Further experiments showed that hCG-increased expression of COX-2 was attenuated by knock down of C/EBPα in human granulosa cells (**Figures 4N–P**). Characterization of C/EBPα showed the same pattern of expression. These results suggest that C/EBPα plays an important role in hCG-induced COX-2 expression in human GCs.

### cAMP Modulates the Activity of C/EBPα to Stimulate COX-2 Transcription *In Vitro*

Cognate receptor of LH is G-protein coupled receptor that is predominantly mediated by activation of adenylate cyclase and cAMP-dependent mechanisms in ovarian follicle growth and maturation (31). As C/EBPα is identified with both constitutive and cAMP inducible activities (32), we next examined whether COX-2 expression was mediated through a cAMP- regulated pathway.

**Figure 5:**
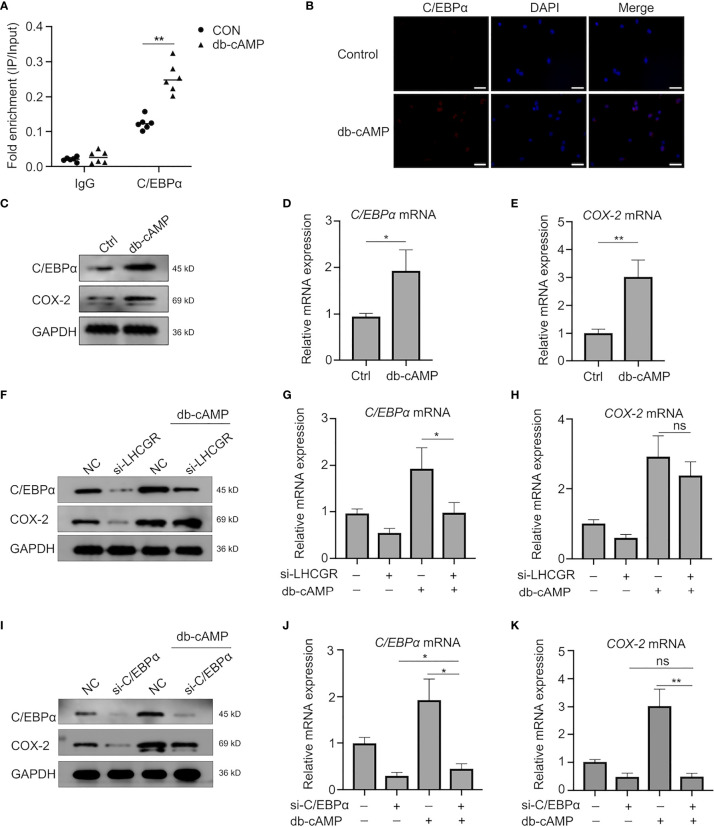
Transcriptional activity of C/EBPα is regulated in a Cyclic AMP-Independent manner. **(A)** KGN cells were left untreated or stimulated with db-cAMP. ChIP assays were performed using anti-C/EBPα antibody or isotype control antibody (IgG). qRT-PCR was used to determine C/EBPα occupancy at the potential biding site under the conditions tested. **P <0.001 (Student’s t-test). **(B)** db-cAMP-treated (24 h) human GCs were analyzed by immunofluorescence to identify the subcellular localization and protein expression levels of C/EBPα (red). Nuclei were stained using DAPI (blue). Magnification: ×100. Scale bar, 50 μm. **(C–E)** Western blotting and qRT-PCR analysis of indicated genes and protein in human GCs after treatment with db-cAMP. *P < 0.05, **P < 0.001 (Student’s *t* -test). **(F–H)** Human GCs were transfected with negative control siRNA or LHCGR siRNA and then treated with 1 mM db-cAMP for 24 h. The expression of indicated genes and protein was analyzed by qRT-PCR and Western blot. ns, no significance, *P < 0.05 (ANOVA). **(I–K)** The protein and mRNA levels of C/EBPα and COX-2 in human GCs, which were treated with 1 mM db-cAMP for 24 h following exposure to siRNAs against C/EBPα. ns, no significance, *P < 0.05, **P < 0.001 (ANOVA).

First, ChIP-PCR assay was conducted to clarify the enrichment of C/EBPα bound to the COX-2 promoter **(** **Figure 5A**). The interaction was also confirmed by results obtained in KGN cells treated with db-cAMP (24 h), indicating that C/EBPα directly binds to the promoter of COX-2 gene to regulate its expression in a cAMP-dependent manner.

To further determine the role of cAMP, human GCs were treated with db-cAMP *in vitro*. The observations showed that db-cAMP improved C/EBPα expression in the nuclei of human GCs as well **(** **Figure 5B**). Treatment of human GCs with db-cAMP for 24 h significantly induced the expression of C/EBPα and COX-2 (**Figures 5C–E**). Indeed, knockdown of LHCGR not only decreased the expression of C/EBPα and COX-2 in basal treatment, but it also exerted a significant inhibitory effect of cAMP-induced expression of C/EBPα and COX-2 (**Figures 5F–H**). Additionally, knockdown of C/EBPα counteracted the cAMP-induced COX2 upregulation (**Figures 5I–K**). Here, we documented that C/EBPα and COX-2 may be induced by LHCGR signaling in a cAMP-dependent manner in granulosa cells. Furthermore, cAMP can promote the transcriptional activity of C/EBPα.

## Discussion

**Figure 6:**
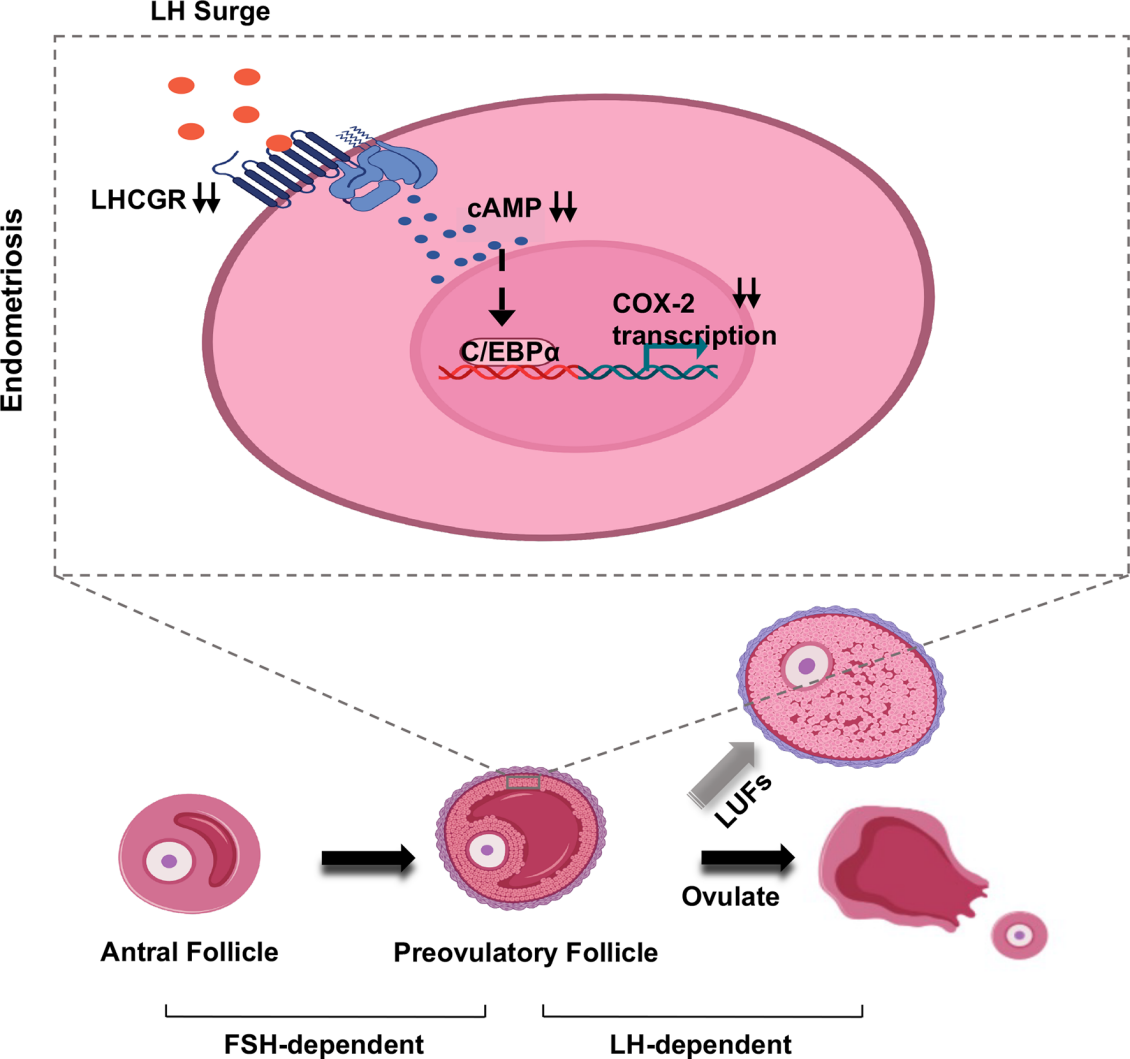
Model of LUFs in endometriosis. Schematic depiction of the effect of LH signaling in preovulatory granulosa cells in endometriosis. During midcycle LH surge, attenuated LHCGR deactivating C/EBPα in a cAMP-dependent manner, then the transcription of COX-2 was repressed in granulosa cells. Ultimately, inducing ovulation failure and oocyte trapped in CLs.

To date, the underlying molecular mechanisms involved in endometriosis-related LUFs remain largely elusive. COX-2 and its major derivative product, PGE2, are recognized to be indispensable factors in the formation of LUFs (9, 33). In this study, we found deceased LHCGR expression in GCs of mice model. The dysfunction may further result in inactivation of cAMP-dependent C/EBPα, which severed as a key transcription factor to regulate COX-2 activation (**Figures 6**). We demonstrated that endometriosis was associated with LUFs because of impaired ovulation function and partially unveiled the underlying mechanism.

LUFs has long been associated with endometriosis in primates (34), rodents (35), and humans (4). In surgical-induced EMs mice, we clearly observed evidence of ovulatory dysfunction due to unruptured follicle which has already developed to preovulatory stage. Although previous studies have claimed that inhibitors (36) and environmental endocrine disruptors (37) may play an important role in impaired ovulation, dysfunctional gene expression in patients with endometriosis draws focus on the expression of LHCGR (16). It is indispensable for granulosa cells to acquire the ability to respond to gonadotropin in follicle differentiation and maturation. As folliculogenesis proceeds, the dominant follicle acquires much higher expression of LHCGR, a gonadotropin-induced G protein–coupled receptor, to allow it to promote ovulation in response to LH (38). In this study, superovulation was initially induced by a standard dose of gonadotropin (5 IU), however, when a lower dose of hCG (2.5 IU) was administered more failed ovulation was observed in EMs mice. It was a matter of interest that the increased unruptured follicles were not found in the sham-operated mice. These results demonstrate that endometriosis reduces GC response to LH, which normally peaks before ovulation. Undoubtedly, the induction of the LHCGR in granulosa cells is a key step in reproductive physiology. Endometriosis is an estrogen-dependent chronic inflammatory condition that affects women in their reproductive period. The local intrafollicular environment and local environment of peritoneal fluid are immunologically dynamic and links the reproductive and immune systems. Alterations in ovarian follicle morphology and function have been documented in affected women. Nevertheless, we documented that the expression of LHCGR was decreased in EMs granulosa cells 48 h after PMSG in this study and it may be a key mediator of endometriosis-associated LUFs. These observations concurred with previous study indicating reduced expression of LHCGR is a key observation in cases of LUFs (39). It is further confirmed by the results obtained in *Lhcgr* knockout zebrafish showing increased unruptured follicles after LH surge (40). Furthermore, the administration of hCG during gonadotrophin ovulation prevents or treats LUFs (41), while a lower dose of hCG may induce LUFs (42). It seems that not only an adequate decrease in intrafollicular prostaglandin but decreased LH or LHCGR responsiveness contributes to the occurrence of LUFs as well. Taken together, it is possible that endometriosis induces attenuation of LHCGR during folliculogenesis. Although the follicle can develop to the preovulatory stage in a follicle-stimulating hormone-dependent manner, the endometriosis-associated pathological states result in decreased responsiveness of granulosa cells to LH peak and subsequently lead to the occurrence of unruptured follicle.

Following activation by LH, LHCGR interacts with a heterotrimeric G-protein (αγβ), generally Gs, that leads to increased intracellular biosynthesis of cAMP (43). Persistent cAMP from internalized LHCGR contributes to transmitting LH signals inside follicles and ultimately to the oocyte (44).Moreover, inactivating mutation of LHCGR has been identified in some women, although follicles of ovulatory size develop fail to ovulate due to decreased cAMP levels (45). In a word, the LHCGR-provoked cAMP, which spreads throughout the follicle is critical to identify the mechanisms involved in the pathogenesis of unruptured follicles, especially after LH surge. Previous studies have originally confirmed that cAMP signaling can increase the transcriptional activity of cAMP-response element-binding protein (CREB) (46), but recent researches provide compelling evidence that C/EBPs also serve as cAMP-responsive transcription factors due to their functionally cAMP-inducible activities (47). Occupying specific cis-elements in the cAMP response unit (CRU), C/EBPα has proved to play a critical role in this process (48). In this study, we found that the attenuation of C/EBPα in endometriosis GCs and a previous study had clarified C/EBPα loss may cause infertility due to LUFs (49). Our results further demonstrated that both hCG and db-cAMP can strongly induce the expression and transcriptional activity of C/EBPα, and the hCG-induced expression can be eliminated by LHCGR knock-down. Thus, it is possible that C/EBPα is hormonally regulated in the ovary and plays an important role during ovarian follicular development and ovulation.

Ovulation is a complex process initiated by the preovulatory LH surge that activates the signal transduction cascades and provokes the expression of numerous endocrine factors. More particularly, many studies have highlighted the important role played by the gonadotrophin-dependent induction of COX-2, which is a key enzyme required for prostaglandin synthesis in the periovulatory follicles (50). In fact, using non-steroidal anti-inflammatory drugs (NSAIDs) would lead to an increase in LUFs in juvenile idiopathic arthritis (JIA) patients due to the effect of inhibition of cyclooxygenase (28, 51). Moreover, animal study has revealed that selective COX-2 inhibitor is a more potent inducers of LUFs (28). Apart from eutopic and ectopic endometrium (52), abnormal expression of COX-2 is also found in cumulus cells of infertile women with endometriosis (53, 54). Our experiments showed that COX-2 expression was decreased in granulosa cells of endometriosis mice. Both C/EBPα and C/EBPβ are expressed in granulosa cells, and are dynamically initiated by LH and hCG to regulate genes that control luteinization and ovulation (55, 56). Although C/EBPβ is the known major regulator of the COX-2 gene, C/EBPβ-deficient ovaries lack corpora lutea and fail to down-regulate expression of COX-2 (32). Therefore, we can speculate that C/EBPα may be involved in this critical progress in ovary. It is well established that the expression of C/EBPα is under the positive control of hCG (57), furthermore, C/EBP-α could serve as a factor mediating COX-2 expression and PGE2 production (58). As C/EBPα gene deletion has resulted in moderately reduced ovulation in mice (49), we further investigated whether C/EBPα is involved in the effects of hCG and cAMP on COX-2 expression in human GCs. Our results indicated that both cAMP and hCG stimulation of COX-2 was eliminated by knock-down of C/EBPα. Using immortalized human granulosa cells, KGN, we further presented molecular and functional evidence that C/EBPα is responsible for regulating COX-2 expression by directly modulating transcriptional activation.

Here, we provide evidence that attenuation of LHCGR in granulosa cells is involved in the increased incidence of LUFs in surgical-induced endometriosis mice. In an *in vitro* cell model system of human granulosa cells, we identify a previously unappreciated role for LHCGR activating transcription factor C/EBPα in a cAMP-dependent manner to sustain COX-2 expression that is necessary for mature follicle rupture and ovulation. We showed molecular and functional evidence that reveals GC dysfunction for the LHCGR as a central mediator of COX-2 expression and may result in LUFs in EMs. Clinical studies and samples acquired from patients are needed in further study to dissect the pathophysiology of this enigmatic syndrome.

## Data Availability Statement

The original contributions presented in the study are included in the article/supplementary material. Further inquiries can be directed to the corresponding author.

## Ethics Statement

The studies involving human participants were reviewed and approved by the Institutional Review Board of Zhongnan Hospital of Wuhan University (No. 2018047). Written informed consent for participation was not required for this study in accordance with the national legislation and the institutional requirements. The animal study was reviewed and approved by the Institutional Animal Care and Use Committee of Wuhan University (Approval No. WP2020-08005).

## Author Contributions

TG and YS conceived the study, performed the experiments, processed the data and wrote the manuscript. YC and LC processed the data and revised the manuscript. ZH, MZ, and LM collected the clinical samples. YZ conceived the study, contributed to the study design and final approval of the version to be submitted. All authors listed have made a substantial, direct, and intellectual contribution to the work and approved it for publication.

## Funding

This work was supported by the National Key Research and Development Program of China (No. 2020YFA0803900) and the National Natural Science Foundation (No. 81771543).

## Conflict of Interest

The authors declare that the research was conducted in the absence of any commercial or financial relationships that could be construed as a potential conflict of interest.

## Publisher’s Note

All claims expressed in this article are solely those of the authors and do not necessarily represent those of their affiliated organizations, or those of the publisher, the editors and the reviewers. Any product that may be evaluated in this article, or claim that may be made by its manufacturer, is not guaranteed or endorsed by the publisher.

## Abbreviations

C/EBPα, CCAAT/enhancer binding protein-α; cAMP, cyclic adenosine monophosphate; ChIP, chromatin immunoprecipitation; CL, corpus luteum; COCs, cumulus–oocyte complexes; COX-2, cyclooxygenase-2; db-cAMP, dibutyryl cyclic-AMP; EGF, epidermal growth factor; GCs, granulosa cells; hCG, human chorionic gonadotropin; ICSI, intracytoplasmic sperm injection; IVF, *in vitro* fertilization; JIA, juvenile idiopathic arthritis; LH, luteinizing hormone; LHGCR, luteinizing hormone/choriogonadotropin receptor; LUFs, unruptured follicle syndrome; NSAIDs, non-steroidal anti-inflammatory drugs; PGE2, prostaglandin E2; PMSG, pregnant mare serum gonadotropin; siRNA, Small interfering RNA.
